# The Evolution of Balanced Scorecard in Healthcare: A Systematic Review of Its Design, Implementation, Use, and Review

**DOI:** 10.3390/ijerph191610291

**Published:** 2022-08-18

**Authors:** Frida Betto, Alberto Sardi, Patrizia Garengo, Enrico Sorano

**Affiliations:** 1 Department of Industrial Engineering, University of Padua, 35122 Padua, Italy; 2 Department of Management, University of Turin, 10124 Turin, Italy

**Keywords:** balanced scorecard, healthcare, performance measurement, literature review

## Abstract

During the last few years, the interest in performance measurement increased within the healthcare sector. Due to the COVID-19 pandemic, healthcare systems needed to boost performance measurement systems to become more resilient and improve their capability in monitoring key performance indicators. Since the 1990s, the Balanced Scorecard (BSC) model has been widely used among private and public organizations as it is the most adopted model to measure performance. The current paper aims at understanding the evolution of BSC in healthcare. The systematic literature review has been carried out by searching keywords according to PRISMA guidelines. By analyzing papers through one classification of BSC adoption phases, the results reveal that studies focused mainly on the BSC design process, rather than BSC implementation, use, or review. However, there is no agreement about the perspectives to be adopted in healthcare. Concerning BSC implementation and use, on one side especially leadership, culture and communication enable the BSC implementation. On the other side, monitoring and strategic decision-making are the most widespread objectives for using BSC. Concerning BSC review, however, the paper highlights a need for additional research. Finally, the paper provides further research opportunities concerning the phases suitable for implementing a BSC in healthcare.

## 1. Introduction

During the last two years, healthcare systems needed to develop resilience to cope with the COVID-19 pandemic [1], control resources, and align targets to their mission in order to continue to effectively deliver care [2].

For many years, pushed by international public reforms [3,4,5,6,7,8,9] or national accrediting bodies [10], healthcare systems and organizations have developed performance measurement systems to improve the efficiency and quality of health care [11,12,13,14,15,16,17,18,19,20]. During the last two years, the pandemic outbreak put even more pressure on the necessity to monitor the internal processes, patient flows, etc., in order to support the efficiency and quality of delivered care services [21,22,23]. The monitoring of key performance indicators was essential in meeting organizations’ objectives [24]. Moreover, the COVID-19 pandemic made clear the necessity to gain real-time information about patients in the department, number of attendances, etc. in order to adapt the organization to the required changes [25].

During pandemics, organizations need to mainly lead the data useful to manage people and patients [26,27]. In that context, such in other previously analyzed contexts (see [28]), the strategy can be defined as an entrepreneurial strategy [29] where the leader, in this case, the strategic task force, takes decisions autonomously. Consequently, organizations need to align their strategic objectives with the day-to-day operations by creating or adjusting their monitoring system to manage organizations and deliver care.

In this context, performance measurement systems play an essential role. Performance measurement is a “process of quantifying the efficiency and effectiveness of an action” [30] in order to monitor it or to “align unit goals with the organization’s strategy” [31].

One model for measuring performance and supporting strategy implementation is the Balanced Scorecard (BSC) [31,32]. Since the 1990s, reforms at the international levels are asking for new management models for measuring and monitoring performance [10,33]. BSC has become a tool supporting the alignment of organizations’ mission, vision and strategy to action by leveraging on performance measurement. The BSC “translates a company’s strategy into specific measurable objectives” and, in this way, the core of the BSC is the strategy and vision of the organization and not control [32]. A few years later, Kaplan and Norton [34] and Kaplan [35] deepened the design of BSC for nonprofit and governmental settings. Traditionally, a BSC model is a “balanced” set of financial and non-financial measures that gives information from four perspectives, i.e., financial, customer, internal business and innovation and learning (or learning and growth) [32].

Nowadays, the Balanced Scorecard is widely spread in healthcare organizations [36,37]. In the last years, private healthcare systems reached high costs, thus costs and value measurement are essential [38]. Additionally, for many years, public health organizations have been pushed to apply effective management systems to measure performance [39].

However, issues related to BSC adoption have been still underlined by literature, as described below.

In order to investigate the BSC research, the authors adopt a recognized framework developed by Bourne et al. [40]. In their study, Bourne et al., identified from previous literature three main phases of performance measurement system implementation, i.e., design, implementation and use; besides, they underlined also the essential role of updating and reviewing processes of measures and targets. Even if they built the classification basing the study on the manufacturing sector, the framework is not context-dependent. By adopting Bourne et al. [40]’s framework, the authors analyzed the BSC implementation process as a whole.

Moreover, in an uncertain and continuously evolving environment, the design of the adopted BSC needs to be updated to cope with the external changes and the target and performance indicators need to be reviewed to be aligned with the organization’s strategy. The traditional BSC perspectives [32] are not always able to catch the new needs, such as the environmental dimension [41], or integrated care and humanization [42]. As a consequence, specific redesign of BSC perspectives aligned with the organization’s strategy and planned updating processes for measures and targets should be carried out by the organizations.

Concerning the implementation of BSC, since the beginning of the 2000s, several authors have investigated not only the drivers but also the barriers to implementing BSC [36,41,43,44]. Inamdar et al. [45] described the challenges faced by applying the Balanced Scorecard in a healthcare organization (e.g., the need for obtaining executive time and commitment, making the scorecard simple and easy to use). Recently, other literature reviews [46,47] emphasized the importance of stakeholder engagement. As drivers of successful BSC implementation, some authors listed trust, leadership support, etc. However, further research is required to explore the drivers of BSC implementation such as the involvement of patients in the BSC reporting and development strategies that overcome the simply customer satisfaction questionnaires [46]. Notwithstanding the increasing interest in patient-centered care or community building, a recent review [46] identifies that, even though needed, “the patients are not engaged to support patient and family-centered care”.

Regarding the use of BSC among healthcare institutions, only a few research explicitly emphasizes how organizations use BSC [42,48] according to the adopted classification of BSC usage [49]. Related to the public sector, an empirical study highlighted that organizations have to assess several mandatory targets, they used BSC for legitimacy seeking [48]. Another case described how organizations have sought to use BSC for strategic decision-making and monitoring internal processes [50]. However, the studies available are not enough to understand how healthcare organizations are using BSC (only a few papers declare explicitly how they use BSC in day-to-day activities). Consequently, further investigation is needed to shed light on this research field.

Due to the current events caused by COVID-19 pandemics, the above-mentioned gaps related to BSC design, implementation, use and review, need even more urgent answers for deepening how to support healthcare organizations in assessing performance.

To address the research gaps, the paper aims at answering the research question below: *How has research on the design, implementation, use, and review of BSC in healthcare settings evolved over the years?*

In light of the development plans required for healthcare systems resulting from the COVID-19 pandemic [51,52,53], the study highlights the state of the art of the BSC model. By mapping the development of BSC over the years it provides two main contributions: it describes how BSC has been designed and implemented in healthcare organizations to favor an efficient and effective use of resources and it provides a comprehensive overview for future empirical research in this area.

Drawing from Bourne et al. [40]’s classification of BSC adoption phases, the paper systematically reviews literature, without limiting the timeframe, to analyze the perspectives adopted in BSC design, the barriers and drivers to implement BSC, the several uses and the review processes of the BSC. By providing insight into the BSC evolution in healthcare, the findings could offer avenues for future research both in the academic world and also among policy-makers who could become aware of how healthcare organizations use BSC.

The paper is structured as follows. Section 2 deepens the methodology adopted for the review. Section 3 reveals the findings developed after the data analysis and Section 4 discusses them to provide the avenues for future research. Finally, Section 5 provides the main conclusion of the review and the research limitations.

## 2. Materials and Methods

To strengthen the transparency and rigor of the review, the research process has been driven by the Preferred Reporting Items for Systematic Reviews and Meta-Analyses (PRISMA) methodology [54,55]. PRISMA is an accepted approach developed in 2005 that provides a checklist to guide systematic literature reviews [54,55].

In line with the above-mentioned checklist [54,55] and other recent systematic reviews based on PRISMA [47,56], the following section deepens the review’s phases to select and analyze the selected articles.

### 2.1. Eligibility Criteria

According to the research objective, i.e., to investigate the evolution of the Balanced Scorecard in the healthcare context, the following criteria have been adopted:

- Article characteristics: papers must be written in English and published in scientific journals. The authors’ objective is to analyze the evolution of the BSC in healthcare, thus the involved studies must be mature and approved by the scientific community to ensure a deeper comprehension of their results. Including only scientific journals, however, could introduce an academic bias.
- Topic: papers must be focused on the Balanced Scorecard to improve organizational performance of the system, healthcare organization or healthcare service. Included studies have to incorporate the BSC concept as evaluated by Kaplan and Norton [34]. Moreover, included studies must emphasize at least one of the four phases of the BSC, i.e., BSC design, implementation, use, or review [40].
- Typology of healthcare setting: papers do not must be focused on a specific setting (e.g., hospitals, health systems). To map the BSC evolution in healthcare, it is noteworthy indeed also to evaluate the evolution of research interest in the different settings related to the healthcare context.
- Research methodology: according to PRISMA guidelines, selected papers can develop either empirical research or a literature review. Including both review and empirical papers allows to include record findings of previous literature reviews related to BSC in healthcare (see PRISMA flow diagram [55]).

Consequently, the following sets of papers were excluded:

- Papers not focused on healthcare settings, such as hospitals, primary services, and local health authorities. Thus, papers focused on other settings referred both to healthcare (such as healthcare supply chains) or to other settings (manufacturing, oil and gas, etc.) are not included in the analysis.
- Papers not focused on BSC as a model for measuring performance or as a strategic tool.
- Papers not focused on the BSC adoption phases, i.e., design, implementation, use and review according to Bourne et al. [40]’s research.

### 2.2. Information Sources and Search Strategy

The records have been selected by searching on Scopus, Web of Science (WoS), and PubMed electronic databases. The identification via databases was carried out in April 2022. To map the evolution of the Balanced Scorecard in the healthcare context, the selection was not limited to a fixed time frame.

According to the database, each search string selects records by keywords (Scopus, WoS) or title (PubMed). PubMed string has been limited to the title for narrowing the bias related to the identification of records (e.g., papers focused on clinical evaluation of specific patients’ diseases) and consequently identifying only records consistent with the review aim. Moreover, each search string (see Table 1), limits the records to papers only written in English. The limitation of the English language was carried out in all the search strings to identify only papers that can be understood by the research team.

Table 1 provides evidence related to the strings used for selecting the dataset.

As Table 1 reveals, the terms used in the search strings highlighted the two main topics of the review: the balanced scorecard and the healthcare setting.

Regarding the balanced scorecard, the selected words are “balanced scorecard”, “balanced score card” (it is a less common way to write BSC, although incorrect), and BSC (the acronym of balanced scorecard).

Regarding the healthcare context, the selected words are health (as catches also health system, health facility, etc., but it introduces several biases such as works related to health conditions, health finance, etc.—see next section for more details), hospital, hospitals, hospitalization/hospitalisation. The last four words have the same root word; however, they have been made explicit in order not to introduce another bias related to hospitality (which refers mainly to the tourism context).

### 2.3. Study Selection and Data Collection Process

According to the PRISMA flow diagram [55], the article selection process develops into identification, screening, and eligibility steps in order to identify the papers included in the review.

Each step was performed several times by the authors. The record search process has been reiterated several times as it needed to be revised and discussed among the research team. Once the objective of the review was identified, the keywords were discussed and selected only after testing the search strings. Then, the screening and eligibility steps were developed together with the research team in order to identify effective exclusion criteria.

**Figure 1:**
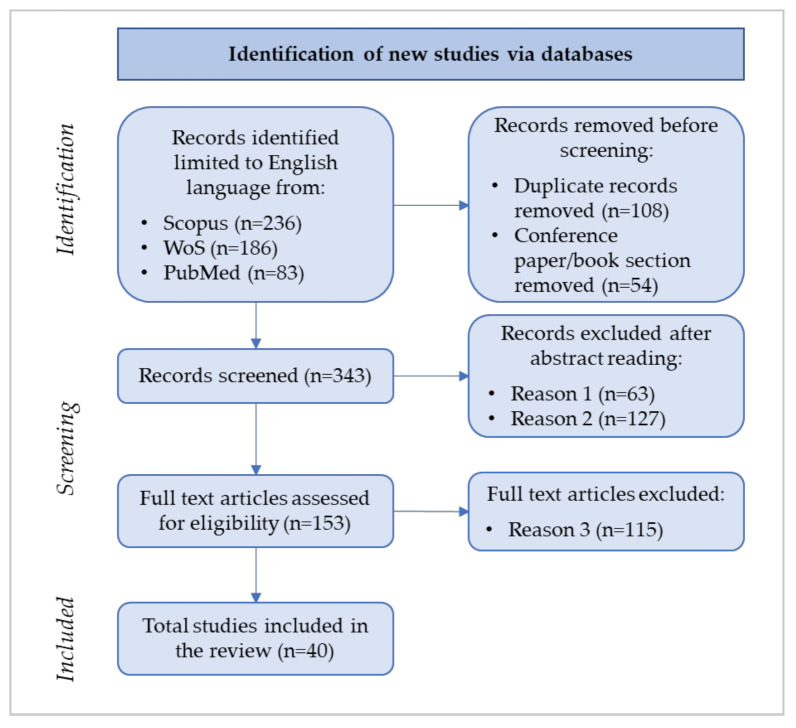
Flow diagram of the review.

Figure 1 shows the review’s flow diagram consistent with the PRISMA guidelines (last access: May 2022).

As Figure 1 illustrates, during the identification phase of the flow diagram 505 records were firstly sorted based on the search strings, and then, after duplicate and conference/book removal, 343 records were selected.

The abstract reading, during the screening phase, was carried out according to the exclusion criteria selected by the research team. According to the exclusion criteria described in Section 2.1, the first exclusion criterion (i.e., Reason 1 in Figure 1) led to the removal of 63 records; the rationale for this criterion refers to the bias introduced mainly by the keyword “health*” embedded in the search strings (as explained in Section 2.2) because it includes non-healthcare-related articles in the 343 identified records (e.g., banking, wastewater, petroleum, manufacturing, etc.).

The second exclusion criterion, i.e., Reason 2 in Figure 1, refers to the papers that do not focus on the balanced scorecard as a strategic tool to enhance organizational performance. Table 2 lists the number of papers excluded according to Reason 2.

After the abstract reading, in line with the PRISMA guidelines [57], the screening phase proceeded with the full-text reading of the 153 papers. The rationale of the third exclusion criteria is consistent with the framework, explained in the following section, adopted to analyze data.

The 40 selected papers deepen BSC design, implementation, use, or review and enable the research team to answer the research question.

### 2.4. Data Items and Framework Adopted

The analysis of the 40 papers was carried out by gathering information related to:

- Publication year;
- Country where the empirical research was developed, or, in the case of conceptual/review paper, the country of the corresponding author;
- The methodology adopted: case study, survey, review, etc.;
- Typology of service: private, public, no profit;
- Unit of analysis: health system, health organization/hospital/health facility, department, or specific care service.

**Figure 2:**
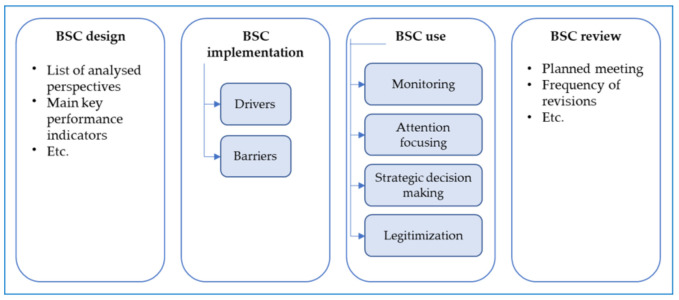
The framework adopted for data analysis.

The above-listed items were used to map the evolution of BSC research in healthcare. Then, to deepen the BSC pathways in healthcare the research team adopted the framework displayed in Figure 2.

Figure 2 reveals the framework adopted to analyze papers. As BSC literature outlines, there are four phases in the BSC adoption [40,58]:

- Regarding the BSC design, the analysis aims at emphasizing the adopted perspectives and the main key performance indicators used;
- Regarding the BSC implementation, the focus is on the drivers and barriers that push or hinder the implementation of BSC;
- Regarding BSC use, the research team adopted a highly cited work that distinguished BSC use in monitoring, strategic decision making, attention focusing, and legitimization, also recently adopted in an empirical study focused on BSC in a hospital [42];
- Regarding the BSC review, in line with [40], the review process includes a set of mechanisms for reviewing targets and standards and processes for a periodical review of the set of measures adopted.

Drawing from Figure 1, the 40 selected papers were analyzed and the main findings are presented in the following section.

## 3. Results

The findings reveal how BSC research in healthcare has evolved across the years. The first section describes the results according to the criteria explained in Section 2.4, then the analysis deepens the description of the results in line with Figure 2.

### 3.1. Descriptive Analysis

The 40 selected papers are published in scientific journals belonging to either “business, management and accounting” or “medicine” research areas (Table 3). This is consistent with the inclusion criteria used to select papers.

The following subsections show the classification of papers by the selected data items (see Section 2.4).

#### 3.1.1. Publication Year

According to Section 2.4, the publication years have been investigated. Notwithstanding the huge timeframe between 1999 and 2022, Figure 3 illustrates the trendline continues to increase over the years (see red line). 

**Figure 3:**
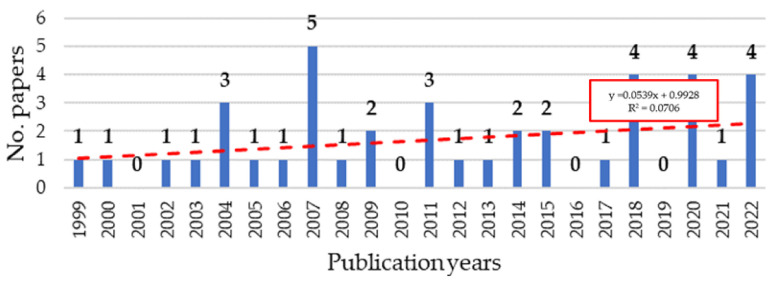
Distribution of papers by year.

The linear trendline is based on the linear regression model solved by the ordinary least square (OLS) method [59]. In Figure 3, it has been pointed out that the regression equation and R^2^ represent a goodness-of-fit for the linear regression model. The more R^2^ is close to 1, the more the model fits the data. In this case, R^2^ is very low, thus the correlation between the timeframe and the published paper is weak. However, the linear trendline shows a positive slope, consequently, the interest in the investigated topic is increasing.

#### 3.1.2. Countries

To map the evolution of papers, it is worth assessing in which countries articles have investigated BSC in healthcare.

The countries were grouped by country areas:

- Asia includes empirical research developed in Afghanistan (2), China (3), India (1), Indonesia (1), Iran (1), Malaysia (2), Thailand (1), and Vietnam (1);
- Australasia includes empirical research developed in Australia (3) and New Zealand (1);
- Europe includes empirical research developed in Germany (1), Greece (1), Hungary (1), Italy (5), Netherlands (1), Portugal (1), Spain (2), and Sweden (2);
- North America includes empirical research developed in Canada (3) and the USA (4);
- UK includes empirical research developed in the UK (5).

**Figure 4:**
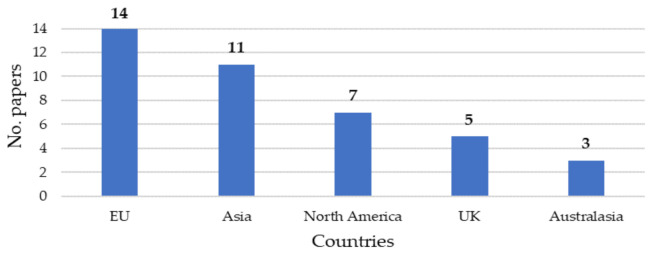
Distribution of papers by country areas.

Figure 4 illustrates the number of published papers classified by geographical areas. Papers based on empirical studies developed in Europe represent the largest set.

**Figure 5:**
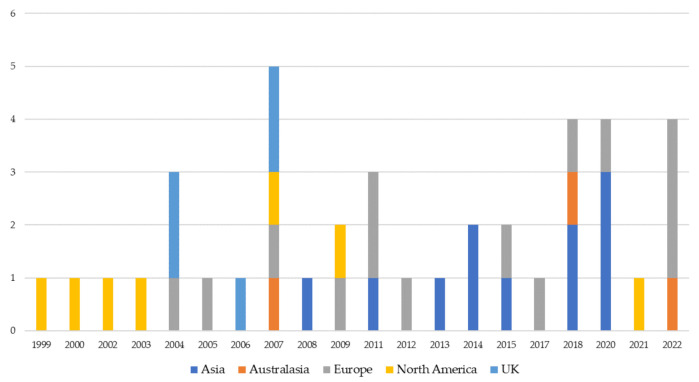
Distribution of papers by country areas and years.

To deepen the analysis, the authors investigate also the papers published in the five geographical areas over the years (see Figure 5).

As shown in Figure 5, there is a prevalence of studies placed in Asia and Europe in the last ten years (85% of papers analyzed in 2012–2022 were placed in Asia and Europe). Whilst analyzed papers placed in Europe have been published since 2004, those placed in Asia started their evolution four years later showing an increasing interest in BSC in the 2013–2020 timeframe (57% of papers analyzed in that timeframe were placed in Asia).

Finally, it is worth noting the prevalence of papers related to the UK and North America is decreasing. As Figure 5 depicts, in the timeframe 1999–2009 the analyzed studies placed in the UK and North America represent 64.7% of the total, while from 2010 to nowadays they represent only 4.3% of the total. Their evolution began before the others (e.g., from 1999–2003 there are only North American studies) and in the last few years, the interest in the topic evolves towards specific applications. USA and UK before applying the exclusion criteria reached the highest numbers of published papers (e.g., in Scopus 39 related to the USA and 19 to the UK), though most of them have been excluded during the paper selection process according to the research questions of this study. However, further clarification is required. As shown in Table 2, most of the excluded papers were related to evaluating human resources, instruments, specific patient flows, etc., thus they have been excluded. This is consistent with the evolution of papers over the years, the UK and mainly the USA began to investigate BSC in healthcare former than the other countries. Now, after more than 20 years, they continue to examine BSC but related to specific processes or uses that overcome the organizational and strategic levels.

#### 3.1.3. The Methodology Adopted to Implement the BSC

The methodology reveals how the authors formulate the research questions and how the empirical study has been carried out. Therefore, to analyze the evolution of papers concerning BSC in healthcare settings, the investigation of the methodologies adopted brings to the understanding of how and where the studies evolve.

Following Yin’s [60] classification of methodologies and adding the review, it is worth noting that the authors investigated BSC in healthcare mainly adopted case study methodology (see Table 4). Conversely, archival studies [61,62] and action research [63,64] have been seldom adopted.

The classification of paper methodologies by country reveals Europe, North America and UK as the country areas where research has been focused mainly on case studies (Table 4).

**Figure 6:**
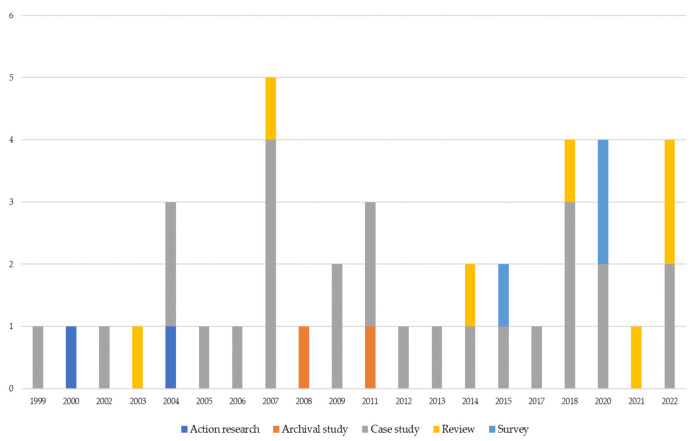
Distribution of papers by methodologies and years.

In addition, to understand the evolution of research methodologies over the years, the graph represented in Figure 6 has been developed. The case study methodology is the most adopted. According to Yin [60], case studies can be used either in explorative research if the research is new and phenomenon driven or to answer questions concerning how and why a research topic develops. Case studies have been adopted in different ways. Oliveira et al. [65] adopted a longitudinal case study in order to understand how BSC is implemented in healthcare settings and whether BSC includes neo bureaucratic traits. In the same way, other authors adopted longitudinal case studies for understanding the evolution of BSC over a fixed timeframe in the same hospital [42]. Case studies have been adopted to investigate the BSC design [45,66,67]. In other cases, participative case studies [50] and action research [63,64] contribute to developing a BSC.

Additionally, surveys and archival studies evaluate the performance gained through the use of BSC [62,68,69] or assess the diffusion of the BSC model [70] within the health system.

Moreover, as the topic is getting mature (the review’s period is more than 20 years), papers based on review [36,46,47,71,72] represent 30.8% of the total in the last five years, whilst before (1999–2016) the papers analyzed based on review were the 11.5% of the total. Finally, even if the selected papers based on the other methodologies are few, surveys are increasing whereas action research and archival studies are decreasing (Figure 6).

#### 3.1.4. Typology of Service

Another item included in the analysis is the typology of service that healthcare organizations or services deliver (Table 5). Four categories have been examined: private service, public service, non-profit organizations (NPO) and non-governmental organizations (NGO). 28 papers considered public services: the majority of paper belongs to Europe, Asia, and the UK where health services are almost everywhere public.

Finally, the other 13 papers belong to private services, NPO, and NGOs. In three empirical studies, the typology of service has not been specified.

#### 3.1.5. Unit of Analysis

**Figure 7:**
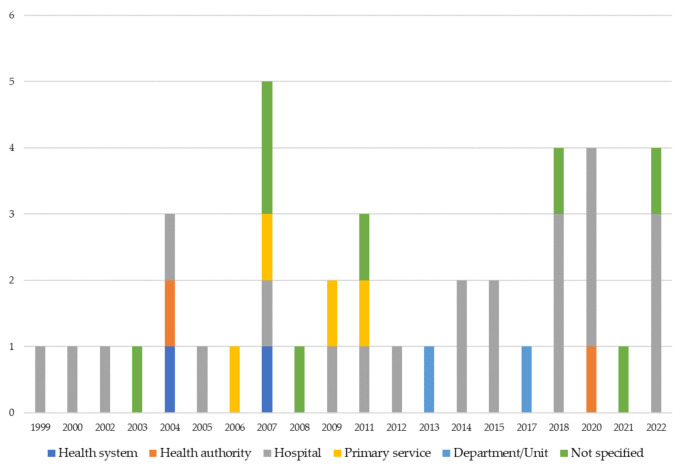
Distribution of papers by units of analysis and years.

Finally, the last investigated item refers to the unit of analysis the selected papers adopted. Table 6 sums up the number of papers that adopted a unit of analysis classified by geographical area. As shown in Table 6, the units of analysis have been classified by macro levels: systems, health authorities, hospitals, primary health services and department units. The results reveal that the hospital level is the most investigated one over the areas (Table 6) and the years (Figure 7). In all the geographical areas of the analysis, the empirical studies investigated mainly the hospital level to analyze BSC in healthcare.

Even by exploring the evolution of units of analysis over the years, the predominance of hospital-level as a unit of analysis remains always clear. The papers focused on hospitals as the unit of analysis represent 55% of the total.

### 3.2. The BSC Phases 

The adoption of a BSC follows four phases [40]. As explained in the BSC, this review analyzes papers by exploring the BSC phases (see Figure 2). 

The subsections below deepen the results related to each phase of a BSC adoption.

#### 3.2.1. BSC Design

Regarding the BSC design phase, the analysis explored the adopted perspectives investigated by different authors in designing the BSC. The analysis sheds light on the most known and adopted perspectives i.e., financial, process, customer and learning and growth perspectives. Even if 30 years passed from Kaplan and Norton’s [32] first work on BSC, the authors still recognized these four perspectives as the most suitable for effective BSC implementation (see Table 7).

The financial perspective has been adopted by most of the studies that focus on BSC design [50,68,69,77]. Empirical research suggests financial indicators based on costs, such as costs of drugs and materials, training costs [68], general system and organization costs [50,63]; expenditures [39,84]; revenues and income [50,84]; efficiency and productivity [63,76]; and some more financial related indicators, such as liquidity and capital [64], financial accessibility and viability [63], net profit margin [39] and capital turnover [83]. 

Financial perspective maintains an essential function in monitoring organizations and services over the years notwithstanding the typology of delivered service (private or public) or the unit of analysis. However, even if healthcare organizations need to fulfil government constraints about budget, expenditures, costs, etc. [50,68,84], the financial perspective is not at the top of BSC perspectives. In line with Kaplan and Norton [34], in public and NPO organizations, the mission is at the top of BSC perspectives [34,68].

Moving from the financial to the process perspective, 18 papers include it in the BSC design. The process perspective includes indicators based on capacity, such as length of stay [68,76], bed occupancy [39,68], bed turnover [68]; quality, such as SDO (i.e., the Italian acronym for the discharge form) quality [50,66], postoperative recovery time and infection rate [83]; efficiency, such as efficient production, distribution and logistics [83], internal efficiency [50]; human resources, such as availability of staff [39,63,66], staff satisfaction [39,63]. 

Moreover, as Table 7 shows, the process perspective named process-related perspectives embodies other perspectives strictly related to processes. For example, emergency areas and emergency services [42] relate to the traditional process perspective but have been highlighted by the authors as the regional government identified emergency areas as a weakness.

In the same way, the customer-related perspective embodies customer, stakeholder satisfaction and workforce perspectives. This perspective has been studied by 20 papers. Indicators concerning customers include patient [39,61,68,76] and, in general, stakeholder satisfaction [67,85], waiting time [83], burden of medical expenses [84], etc. 

Concerning healthcare, several studies [68,71] place customer perspective at the top of BSC perspectives, whereas another focuses on a more specific perspective related to customers, i.e., stakeholder satisfaction [50], and places it at the top.

Although learning and growth is the fourth perspective belonging to the traditional BSC built by Kaplan and Norton [32], a lower number of studies adopted it rather than the other three perspectives above mentioned. The learning and growth perspective refers to non-financial measures (as well as the customer and internal processes perspectives), mainly based on personnel structure [84], employee training [39,76,83], and staff satisfaction [68,83]. It is worth noting that staff satisfaction indicator belongs either to learning and growth [68,83] or to “stakeholder satisfaction” perspective [50] (which includes both employees and patients, which was grouped here under the customer-related perspective set), or to process perspective [39].

“Learning and growth” shed light on skills and competencies the staff have to reach, such as number of studies [68], number of participation in conferences [39], adaptation to new technologies [76,83], training, but also on the satisfaction of staff derived from work [68,83].

Notwithstanding the recognized relevance of this perspective in traditional BSC [32,34] and in BSC built for healthcare, several studies neglect it. Gao et al. [84] mentioned learning and growth as the last of the four perspectives due to low interest in research and teaching processes in the involved Chinese county hospital. In other cases, “learning and growth” has been removed narrowed to the specific healthcare context. For example, Catuogno et al. [50] investigated a research hospital and they removed learning and growth and identified other perspectives, such as “research process” and “stakeholder satisfaction”. The mission of this kind of organization, i.e., research, needs a specific focus on research that has been emphasized by the research process perspective. However, inside it, some belong to “learning and growth” indicators, such as scientific articles.

In addition to the four traditional BSC perspectives, empirical studies on BSC in healthcare analyze others that have been described below:

- Community-related perspective. It groups the perspectives related to community, external environment assessment, and fair access. They consider waiting times [48,86], response to emergencies [86], equity factors [48,61]. The studies that include the community perspective focus on the health system or service and consequently place great attention on population needs [61]. Otherwise, they emphasize the needs for outpatient and inpatient even within healthcare organizations by modifying traditional perspectives.
- Quality-related perspective. It groups the perspectives related to appropriateness and quality [48,66,74], organizational excellence [85], surgical performance [42], humanization [42], and health outcomes [48]. Three papers focus on the appropriateness and quality of the organization. Broccardo [66] includes this perspective in order to evaluate the appropriateness of Diagnosis-Related Groups (DRG) and also the average weight of hospital stay, strictly related to other indicators belonging to the financial perspective [68,76]. In other studies, appropriateness is related to the quality of delivered care, such as chronic and acute care management, and inappropriate use of surgery [48]. In this case, these factors could be associated with the process perspective, as focused on care processes.
- Innovation perspective. Groene et al. [75] identified innovation as the perspective to promote communication, train staff, assess satisfaction, establish regular self-assessment, etc. There, the innovation perspective replaced learning and growth and indeed the strategic objectives are very similar.
- Strategy-related perspective. It groups the perspectives related to the mission and achievement of strategic objectives. Soysa et al. [67] highlight the function of Mission, i.e., to improve Learning and Growth. Other authors renovate the function of mission and strategy function to drive the other perspectives [42,69].
- Efficiency-related perspective. It groups the perspectives related to health improvement and efficiency [48], integration and responsiveness, patient attraction waiting times, capacity for service provision, and environmental performance [41]. For example, Edwards et al. [61] identified capacity for service provision as a perspective that enables health services to procure medicines, equipment, clinical guidelines, etc. Moreover, Chang identified a dimension of efficiency concerning how financial resources need to be spent. This is one case in which the financial perspective has been replaced by another, i.e., efficiency, to evaluate day case rate, length of stay, etc.

**Table 7:** Perspective identified after the analysis.

BSC Perspectives	No. Papers	Main References
*Financial perspective*	23	[31,43,45,46,55,56]
Process	18	[31,42,43,55,62,63]
Emergency areas and emergency service	1	[42]
Integrated care processes	1	[42]
Service provision	1	[61]
Clinical risk	1	[81]
Service modernization	1	[81]
*Process-related perspective*	23	
Customer	15	[39,61,68,77,84]
Stakeholder satisfaction	3	[50,67,85]
Workforce	2	[81]
*Customer-related perspective*	20	
*Learning and growth perspective*	13	[44,54,55,56,62]
Community	4	[44,61,80,86]
External environment assessment	2	[80,87]
Fair access	1	[48]
Overall vision	1	[61]
*Community-related perspective*	8	
Appropriateness and quality	3	[48,66,74]
Organizational excellence	1	[85]
Surgical performance	1	[42]
Humanization	1	[42]
Health outcomes	1	[48]
*Quality-related perspective*	7	
*Innovation perspective*	3	[63,75,85]
Mission	3	[67,69,87]
Achievement of strategic objectives	3	[69,87]
*Strategy-related perspective*	6	
Health improvement	1	[48]
Efficiency	1	[48]
Integration and responsiveness	1	[80]
Patient attraction waiting times	1	[42]
Capacity for service provision	1	[61]
Environmental performance	1	[41]
*Efficiency-related perspective*	6	

**Figure 8:**
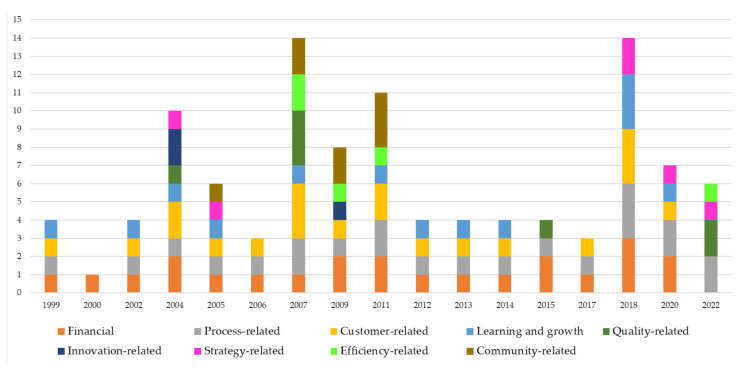
Distribution of papers by BSC perspectives and years.

Moreover, to investigate the evolution of perspectives over the years, the authors deliver the graph in Figure 8. The process and financial perspectives show a prevalence over the other identified perspectives over the years (they represent 39.25% of the total).

Moreover, still a great deal of attention is required to process financial and learning and growth perspectives; those perspectives mainly focused on people are the most neglected by the selected sample of papers. Surely, the lower attention on community related-perspective is consistent with the low number of papers focusing on systems and primary care, as shown in Table 6. On the contrary, the decrease in customer related-perspective sheds light on a lack of focus on human resources.

The design of BSC has been investigated by 34 of the 40 papers. Even though the traditional perspectives of the BSC, i.e., financial, customer, processes and learning and growth, are the most adopted, numerous authors investigated other perspectives over years. In line with the explanation of indicators and purposes of the new perspectives, it is worth noting that they are not completely independent from the four main ones. Some indicators, such as training, length of stay, etc., are in common. Authors that adopt new perspectives shed light on specific facets, for example efficiency to emphasize how the organization use financial resources [42] or community to emphasize how the community perceives the health services delivered [61].

#### 3.2.2. BSC Implementation

Regarding BSC implementation, this study investigates the drivers and barriers identified from the review and summarizes them in Table 8. The items identified are very specific, and often mentioned only in one paper. The authors did not classify them as not losing the meaning provided by the empirical studies.

Regarding the drivers, communication, leadership support and training, were the most emphasized. Communication, as well as meetings to define responsibilities, have been considered both as a driver to develop BSC and in turn as factors that BSC boosts [46,65]. Additionally, leadership has been identified by many studies as a driver for the implementation of the BSC [46,61]. However, if the commitment and the participation are low, leadership act as a barrier. Many studies consider leadership a negative factor because there is a lack of ownership [43], a lack of managers to replace administrators [43] or a lack of champions [41].

In the same way and strictly related to leadership, also organizational culture plays an essential role in the BSC implementation phase. Organizational culture has been analyzed in relation to performance measurement both in private [88] and in public organizations [89]. Here, related to BSC, it has been considered both a barrier and a driver. On the one side, some authors consider it a barrier [41] if there is a command and control culture not prepared to implement a tool to measure performance. For example, in [44], the medical staff perceived the BSC only as a tool for controlling clinicians.

On the other hand, culture is a driver if there is a collaborative culture and people participate in short-term solutions [65], participation is high [80] and people have been trained [43,65] and involved in the BSC implementation process [85].

The results, i.e., the barriers and the drivers of BSC implementation, reveal firstly that only 12 of the 40 papers mentioned them analyzing BSC adoption in healthcare settings. Secondly, the barriers and drivers are represented quite frequently by the same factors. Organizational culture and leadership, for example, can be a driver if the culture is democratic and participative and leadership is transactional (in line with the definition of Bass and Avolio [90]). Otherwise, if there is a command and control culture or a laissez-faire leadership style, BSC could be difficult to be implemented.

#### 3.2.3. BSC Use

To analyze the use of BSC, the classification provided by Henri [49] and implemented in other healthcare studies [42] have been adopted. Henri focused on four main use of BSC. Even if only a few papers explicitly mention the use of BSC, the authors were able to provide, if possible, the analysis of BSC use consistent with Henri’s [49] definition.

In line with Henri [49], there are four typologies of performance measurement use. Firstly, monitoring acts as a diagnostic control (see Simons [91]) and thus BSC measures and reports performance. As recently discussed by Bassani et al. [42], the most widespread use of BSC is monitoring. Given the fact that most of the papers on BSC in healthcare focus on BSC design, monitoring use has been emphasized. However, few studies explicitly mention the BSC use. Pham et al. [69] evaluated the financial and non-financial measures based on BSC to assess performance in specific Vietnamese mountainous areas. Soysa et al. [67] developed a BSC-based scoring system to provide performance measures. Papers focused on BSC design and assessment of performance implicitly make a monitoring use of BSC [67,69,72].

Secondly, attention focusing acts as interactive control. Workers should focus their attention on specific objectives. This BSC by reflecting a focus on specific objectives related mainly to the process dimension. In this review, “attention focusing” use, even not explicitly mentioned (apart from [42]), has been identified in the studies that put great emphasis on process measurement. For example, in [65] the implementation and use of BSC were timed by regular meetings, specific objectives were fixed, and the staff was responsible for its objectives.

Thirdly, strategic decision-making acts in fixing strategic decisions for problem-solving. As shown before, some authors emphasized strategy and mission values and identified them as stand-alone perspectives [42,67,69,87]. Amer et al. [47] recently classified BSC generations and define the second generation of BSCs as those focused on a strategy where users align the objectives to the strategy and mission of the organization. In this way, Bassani et al. [42] and Oliveira et al. [65] argue that BSC is used to align specific objectives to the organization’s strategy.

Lastly, legitimization acts to justify past actions or decisions. Kollberg and Elg [92] in their study emphasize the legitimization use of BSC for information sharing and reporting, while Chang [48] negatively considers legitimization use. In his study, the analysis focused on the NHS performance assessment framework and the relationship between the central government and local health authorities. Chang highlighted that if there is no communication between the central level and peripheries, measurement systems can be used only to legitimize actions and not to improve the systems [48].

**Figure 9:**
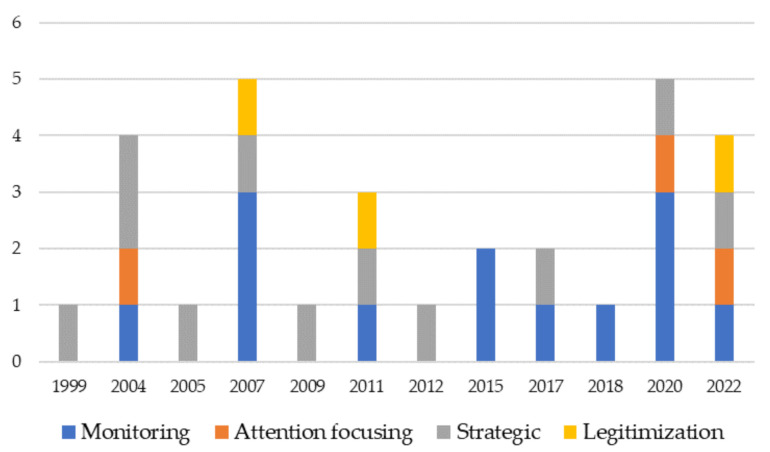
Distribution of papers by BSC use and years.

To map the evolution of BSC over the years, Figure 9 provides a graph of where published papers were distributed by years and BSC use.

Monitoring and strategic use of BSC are the most widespread in line with the traditional use of BSC [32,93].

#### 3.2.4. BSC Revision

The BSC revision implies scheduled revisions of indicators and targets thanks to planned meetings and information-sharing processes. This last phase of BSC adoption has been seldom mentioned in the papers. This is consistent with the great attention on the first phase of BSC, i.e., the BSC design.

Studies focused on the design and implementation of BSC are not enough mature to develop a clear understanding of BSC revision processes.

Only five papers include explicitly also BSC review [41,47,74,75,80,94]. The major focus is on the BSC revision to align the objectives of the indicators to the organization’s strategy and mission [41,47].

## 4. Discussion

The findings reveal that research on BSC in healthcare contexts is still directed toward the BSC design process. The majority of papers (34 of the 40 papers) paid particular attention to the BSC perspectives. In line with Gonzalez-Sanchez et al. [36], the analysis shows that papers focus mainly on the traditional four perspectives, i.e., financial, customer, processes, and learning and growth. Notwithstanding the maturity of the BSC model which emphasizes the balanced nature of the perspectives, financial is still the most analyzed (see Table 7). As a matter of fact, even if the prevalent typology of delivered service is public, the healthcare settings at all levels (systems, organizations, primary services, etc.) need to put great attention to financial indicators such as expenditures [39,84], financial accessibility and viability [63], and net profit margin [39].

This is in line with the monitoring use of BSC, implicitly underlined by many authors [67,69].

However, although the financial perspective remains prevalent over the years, the process-related perspective is rapidly increasing in the number of published papers that analyze it [39,63,66]. This is in line with the “attention focusing” use of BSC, where workers are motivated to achieve specific objectives (adapted from Henri’s definition [49]). Indicators such as bed turnover, post-operative infection rate, etc., describe specific objectives to be achieved at the strategic level but also the operational level within the single service or department.

In addition to the most frequently analyzed perspectives, the review sheds light on the other five groups of perspectives. As highlighted in the Findings section, several indicators match financial, process, customer or learning and growth indicators (e.g., weight of hospital stay belongs to financial [68,76] and community [66] perspectives). The paper highlights that BSC research is still mainly focused on BSC design, and, even if new perspectives emerge to emphasize some strategic objectives of organizations or systems, they are quite similar to the four traditional perspectives. They are often a subset of financial, customer, learning and growth, or process perspectives.

Thus, some future research avenues could be addressed.

Given the emphasis put on the BSC design, future research could analyze why there is no agreement between the perspectives. Even though the indicators are different between organizations and settings, the perspectives are not consistent with a specific healthcare setting. For example, the community perspective has been adopted by healthcare organizations [86,87] and by primary health services [61,80]. At the same time, the community perspective embodies some indicators belonging traditionally to the customer perspective [61].

Concerning barriers and drivers in implementing BSC, the findings shed light on a lack of a standardized way to rank the factors that enable or restrain BSC. Some drivers match each other, for example, communication [46,74], absence of duplicated information and meetings are closely related. Similarly, leadership support can foster process understanding [43], collaborative culture [65] and participation [80]. The same happens with barriers. The lack of organizational culture ready to accept change is closely linked to the perception of BSC as a control tool, to a narrow view of BSC, etc. In addition, papers focus on similar factors, viewing them as barriers or drivers of BSC implementation, such as leadership and organizational culture, depending on how they are perceived. 

Although analyzed by several studies [36,41,61,65], there is a need for further research for identifying a standard way to explicate barriers and drivers of BSC implementation. Thus, future research could focus on management practices that enable BSC implementation at the different organizational levels (i.e., systems, organizations, primary care services, units/departments).

Concerning the use of BSC in healthcare organizations, only a few papers declare the BSC use that the organizations/systems did. Although the review identifies 13 papers concerning monitoring and 11 concerning strategic decision-making use of BSC (in line with the framework provided by Henri [49]), the papers do not explicitly refer to how the organizations use the BSC. The remaining papers, on the other hand, did not reveal even implicitly how organizations use the BSC. Thus, future research can stress more explicitly how diverse healthcare settings use the BSC.

Regarding the last phase of BSC adoption, i.e., BSC review, only a few papers explicitly mentioned it [41,47,74,75,80,94]. Future research needs to focus on this fourth phase, even though during the initial phase of BSC design, the review process is essential, meetings and participation in short-term decisions [65] can favor the implementation of BSC.

Finally, concerning the current situation, where healthcare organizations are coping in the last two years with the COVID-19 pandemic, it would be interesting empirically investigate the different outcomes achieved in the organizations that already implemented a BSC model and in those that used other measurement systems. Given the importance of gaining real-time information during a crisis [26,27], it would be interesting to analyze the effectiveness of BSC in this specific context. In the same way, it would be interesting to understand which BSC measures are useful during a pandemic context to endure quality and efficiency in delivering care. Notwithstanding the positive outcomes derived from BSC implementation and use, as in the review of Amer et al. [47], no studies have been found related to the effects of BSC during pandemics.

## 5. Conclusions

The paper addresses the research question, i.e., how BSC evolved over the years in health settings, by mapping the development of BSC adoption phases [40] over the years in healthcare. Through the review’s framework illustrated in Figure 2, the BSC’s main perspectives, the barriers and drivers in implementing BSC, and the use and review of BSC have been analyzed. Although the prevalent interest in BSC research is on the design, the review analyzes several papers that focus also on the barriers and drivers, and the use of BSC (even not explicitly). However, only a few papers analyze the review process of BSC, by shedding light on the low maturity of BSC in healthcare contexts.

Moreover, the analysis of the review on BSC in healthcare settings, differently from the recent review of Amer et al., [47] reveals that it is not possible to identify three generations of BSC. By adopting the review’s framework (Figure 2), BSC adoption phases [40], i.e., design, implementation, use and review are strictly related. As the findings reveal, for example, a specific focus on process perspective is followed by an increase in the “attention focusing” use of BSC. The liaison is represented by the recognized relevance of leadership support to promote a collaborative culture in implementing BSC.

Although the findings give room for several future research avenues, as discussed in the previous section, the review has some limitations. Concerning methodology, in the review have been selected search strings based on keywords in Scopus and Web of Science and on the title in PubMed. This choice, even if adopted in other reviews [95,96], has limited the results. Consequently, future research could run the search strings by investigating also the title and abstract (regarding Scopus and Web of Science). Moreover, the involvement of academic journals only can introduce an academic bias. Thus, future research could consider also conference papers, books, and grey literature.

Finally, the authors identified BSC as the focus of their research because it has been the most adopted model over the years. However, future research could verify through a literature review what are the other tools/models the healthcare settings use to measure performance, or if they are not adopting tools.

## Figures and Tables

**Table 1:** Search strings (^1^ author keywords (AK); ^2^ keywords plus (KP)).

Scopus Search String	WoS Search String	PubMed Search String
(KEY (“balanced scorecard*” OR “balanced score card*” OR BSC)) AND (KEY (“health*” OR “hospital” OR “hospitals” OR “hospitali*ation*”)) AND (LIMIT-TO (LANGUAGE,”English”))	(AK ^1^ = (“balanced scorecard*” OR “balanced score card*” OR BSC) OR KP ^2^ = (“balanced scorecard*” OR “balanced score card*” OR BSC)) AND (KP = (“health*” OR “hospital” OR “hospitals” OR “hospitali*ation*”) OR AK = (“health*” OR “hospital” OR “hospitals” OR “hospitali*ation*”)) AND English (Languages)	(“balanced scorecard*”[Title] OR “balanced score card*”[Title] OR BSC[Title]) AND (“health*”[Title] OR “hospital”[Title] OR “hospitals”[Title] OR “hospitali*ation*”[Title]) AND (english[Filter])

**Table 2:** Excluded papers according to the second criterion during the screening phase.

Related Topics	No. of Excluded Papers
Measurement systems not explicitly focused on BSC	80
BSC to evaluate clinical pathways, procedures, etc.	12
BSC used for technology evaluation	8
Performance assessment of human resources	5
BSC in education/universities (medical school, etc.)	5
BSC in supply chain management	5
BSC for facility management	3
BSC for policy evaluation	3
BSC for project management evaluation	3
BSC for sustainability	2
BSC use in risk management	1
BSC use in lean management	1
Evaluation of a COVID instrument	1
Total	127

**Table 3:** Scientific journals of the published papers.

Journals	No. Publications
International Journal of Health Care Quality Assurance	4
International Journal of Health Planning and Management	3
International Journal of Productivity and Performance Management	3
Journal of Health Care Finance	3
International Journal for Quality in Health Care, BMC Health Services Research	2
Australian Health Review, Benchmarking, BMC Public Health, BMJ Open Quality, Burns, Cost Effectiveness and Resource Allocation, Expert Systems with Applications, Health Policy and Planning, International Journal of Electronic Healthcare, International Journal of Public Sector Management, Iranian Journal of Public Health, Journal of Healthcare Management, Journal of Accounting & Organizational Change, Journal of Advances in Management Research, Journal of Asian Finance, Economics and Business, Journal of Health Management, Journal of International Medical Research, Journal of Modelling in Management, Measuring Business Excellence, Omega, PLoS Medicine, Shiraz E Medical Journal, Sustainability Accounting, Management and Policy Journal	1

**Table 4:** Methodologies adopted by the selected papers classified by country areas.

Methodology	Asia	Australasia	Europe	North America	UK	Total
Action research	0	0	1	1	0	2
Archival study	2	0	0	0	0	2
Case study	5	2	10	4	5	26
Review	1	1	3	2	0	7
Survey	3	0	0	0	0	3

**Table 5:** Typologies of service of the selected studies classified by country areas.

Type of Service	Asia	Australasia	Europe	North America	UK	Total	Main References
Private	1	1	4	3	0	9	[45,73,74]
Public	9	2	10	2	5	28	[65,66,69]
NPO	1	1	0	0	0	2	[67,68]
NGO	2	0	0	0	0	2	[61]
(Not specified)	0	0	1	2	0	3	-

**Table 6:** Units of analysis adopted by the selected papers classified by country areas.

Unit of Analysis	Asia	Australasia	Europe	North America	UK	Total	Main References
Health system	0	0	1	0	1	2	[48,63]
Total health systems	0	0	1	0	1	2	
Health authority	0	0	0	0	1	1	[43]
Local health authority	0	0	1	0	0	1	[65]
Total health authority	0	0	1	0	1	2	
Acute care hospitals	0	0	1	0	0	1	[75]
Hospitals	5	1	7	3	1	17	[27,43,48,55,56,57,76,77]
Burn center	0	0	0	1	0	1	[73]
Community hospital	1	0	0	0	0	1	[68]
County hospitals	1	0	0	0	0	1	[78]
Policlynics	1	0	0	0	0	1	[79]
Total hospitals	8	1	8	4	1	22	
Health services	0	0	0	1	0	1	[80]
Mental health service	0	0	0	0	1	1	[81]
Primary health service	1	0	0	0	0	1	[61]
Stop Smoking service	0	0	0	0	1	1	[82]
Total primary services	1	0	0	1	2	4	
Department	0	0	1	0	0	1	[50]
Operating room	1	0	0	0	0	1	[83]
Total departments/units	1	0	1	0	0	2	
(Not specified)	1	2	3	2	0	8	

**Table 8:** Drivers and barriers identified from the selected papers.

**Drivers**	**No. Papers**
Communication, Leadership support, Training	2
Reward or incentive systems, Absence of duplicated information, Transparency, Understanding the processes, Collaborative culture, Participation in short-term solutions, Meetings, Commitment, Participation, Organizational culture, Skills	1
**Barriers**	**No. Papers**
Organizational culture	2
Narrow vision, Lack of ownership and accountability, Multi stakeholders’ needs, Lack of managers, Need to work the system, Role of environmental disclosure, Sustainability, Lack of BSC knowledge, Lack of champions, Absence of environmental commitment practices, Lack of policy, Minimal infrastructure, Corruption, Tool for control for medical staff, Resistance to measurement, Low literacy rate, Disconnection between the central government and local health units, Legitimacy seeking, Aligning interests with mission and vision, Selection of indicators, Timely collection of data, Training	1

## Data Availability

Not applicable.
